# Supplementary material for: Preparation and Characterization of Soluble Dietary Fiber Edible Packaging Films Reinforced by Nanocellulose from Navel Orange Peel Pomace
Source: Polymers (Basel). 2024 Jan 24;16(3):315. doi: 10.3390/polym16030315 (PMC10857216; doi:10.3390/polym16030315)
Supplement: Supplementary file 1 [file polymers-16-00315-s001.zip › polymers-2801268-supplementary.pdf]

Supporting Information

Preparation and characterization of soluble dietary fiber edible packaging films reinforced by nanocellulose from navel orange peel pomace

Lili Chen, Yincai Wu, Yuntian Guo, Xiaofeng Yan, Wenliang Liu *, Si Huang *

1. Supporting Experimental Section

The samples' crystallinity index (CrI) was calculate from Segal's empirical formula according to the following equation:

$$\mathrm{CrI} = \frac{I_{002} - I_{am}}{I_{002}} \times 100\% \qquad \text{(S1)}$$

Where, I_{002} represented the intensity of the diffraction peak at 22.6 ° and l_{am} represented the diffraction intensity of the peak in the amorphous region at 18 °.

The apparent shear viscosity and differential shear viscosity of film solutions was evaluated according to the following equation:

$$\eta_a(\dot{\gamma}) = \frac{\sigma}{\dot{\gamma}} \qquad \text{(S2)}$$

$$\eta(\dot{\gamma}) = \frac{d\sigma}{d\dot{\gamma}} \qquad \text{(S3)}$$

where σ was the shear stress (Pa), $\dot{\gamma}$ was the shear rate (s^{-1}), $\eta_a(\dot{\gamma})$ was the apparent shear viscosity (Pa·s), $\eta(\dot{\gamma})$ was the differential shear viscosity (Pa·s).

2. Supporting Figures

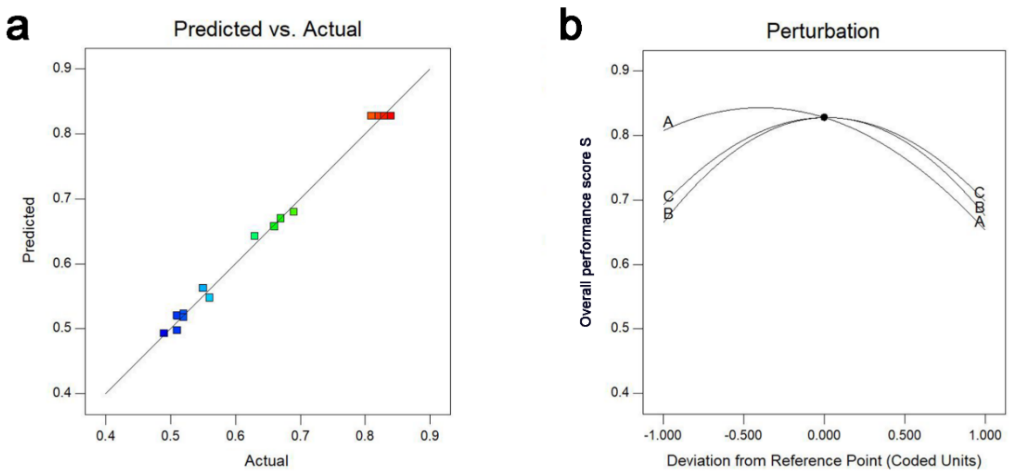

Figure S1. (a) Map of the model residual distribution for the overall performance score; (b) Factor fluctuation effect diagram of overall performance score.

3. Supporting Tables

Table S1 Principal component analysis experimental data of edible packaging film based on dietary fiber from navel orange peel pomace

NO.	TS/(MPa)	EB/(%)	OR/($cc \cdot m^{-2} \cdot 24\ h^{-1}$)	LR/(%)	HP/(N/15 mm)
1	6.73	89.10	0.96	67.97	3.58
2	11.02	128.42	0.87	81.17	3.07
3	6.90	106.68	1.34	74.60	4.64
4	8.49	93.52	1.19	73.90	3.96
5	11.34	59.46	1.09	77.20	3.18
6	6.72	105.38	1.85	75.90	4.79

Table S2 Correlation matrix of performance evaluation parameters of edible packaging film based on dietary fiber from navel orange peel pomace

NO.	TS	EB	OR	LR	HP
TS	1.000	-0.177	-0.554	0.710	-0.807
EB	-0.177	1.000	0.062	0.349	0.214
OR	-0.554	0.062	1.000	-0.008	0.870
LR	0.710	0.349	-0.008	1.000	-0.253
HP	-0.807	0.214	0.870	-0.253	1.000

Table S3 Component loading matrix of principal component analysis of edible packaging film based on dietary fiber from navel orange peel pomace

Component	TS/ (MPa)	EB/ (%)	OR/ ($cc \cdot m^{-2} \cdot 24\ h^{-1}$)	LR/ (%)	HP/ (N/15 mm)
1	0.949	-0.938	0.800	-0.508	0.142
2	0.225	0.183	0.323	0.806	0.761

Table S4 Weight value of performance indexes of edible packaging film based on dietary fiber from navel orange peel pomace

Performance	TS/ (MPa)	EB/ (%)	OR/ ($cc\cdot m^{-2}\cdot 24\ h^{-1}$)	LR/ (%)	HP/ (N/15 mm)
Weight value	0.307	0.195	0.286	0.021	0.195

The weight calculation could be divided into the following three steps: Calculating each index's coefficients in the linear combination of the two principal components was the first step. The load coefficient divided by the characteristic root's square root was the calculation's formula; The second step was to calculate the coefficients for each index in the comprehensive scoring model. The formula for calculation was to multiply the linear combination coefficients by the variance contribution rate, added them together, and then divided them by the total variance contribution rate; The third step was to normalize the coefficients of each index in the comprehensive score model and then obtained their weight values.

Table S5 Overall performance score model fitting analysis

Std. Dev.	0.015	R-Squared	0.9948
Mean	0.65	Adj R-Squared	0.9881
C.V. %	2.29	Pred R-Squared	0.9503
PRESS	0.015	Adeq Precision	29.588

Table S6 Multiple factors combining at S maximum

OSDF	ONCC	Gly	Overall performance score
0.17	4	25	0.843
